# An endogenous lentivirus in the germline of a rodent

**DOI:** 10.1186/s12977-022-00615-2

**Published:** 2022-12-20

**Authors:** Roziah Kambol, Anna Gatseva, Robert J. Gifford

**Affiliations:** 1 School of Biological Sciences, Faculty of Applied Sciences, University Teknologi MARA, 40450 Shah Alam, Selangor Malaysia; 2 MRC-University of Glasgow Centre for Virus Research, 464 Bearsden Rd, Bearsden, G61 1QH Glasgow UK

**Keywords:** Retrovirus, Lentivirus, Evolution, Ecology, Endogenous retrovirus, Paleovirology

## Abstract

Lentiviruses (genus *Lentivirus*) are complex retroviruses that infect a broad range of mammals, including humans. Unlike many other retrovirus genera, lentiviruses have only rarely been incorporated into the mammalian germline. However, a small number of endogenous retrovirus (ERV) lineages have been identified, and these rare genomic “fossils” can provide crucial insights into the long-term history of lentivirus evolution. Here, we describe a previously unreported endogenous lentivirus lineage in the genome of the South African springhare (*Pedetes capensis*), demonstrating that the host range of lentiviruses has historically extended to rodents (order Rodentia). Furthermore, through comparative and phylogenetic analysis of lentivirus and ERV genomes, considering the biogeographic and ecological characteristics of host species, we reveal broader insights into the long-term evolutionary history of the genus.

**Supplementary Information:**

The online version contains supplementary material available at 10.1186/s12977-022-00615-2.

## Introduction

The lentiviruses (genus *Lentivirus*) are an unusual group of retroviruses (family *Retroviridae*) that infect mammals and are associated with a range of slow, progressive diseases in their respective host species groups [1] (Table 1). They are most familiar as the genus of retroviruses that includes human immunodeficiency virus type 1 (HIV-1), but the group also includes viruses that infect a broad range of other mammalian groups. Lentiviruses are distinguished from other retroviruses by several characteristic features, including several unique accessory genes, a characteristic nucleotide composition [2, 3], and the capacity to infect non-dividing target cells [4].

**Table 1:** Reference genome sequences of representative lentivirus species

Species	Host species		Abbreviation/sub-strain	Source^a^
*Exogenous viruses*				
Jembrana disease virus	Gaur	*Bos gaurus*	JDV	NC_001654
Bovine immunodeficiency virus	Domestic cattle	*Bos taurus*	BIV	M32690
Small ruminant lentivirus genotype A	Goats & sheep		SRLV-A	NC_001452
Small ruminant lentivirus genotype B	Goats & sheep		SRLV-B	NC_001463
Equine infectious anemia virus	Domestic horse	*Equus cabalus*	EIAV	M16575
Feline immunodeficiency virus	Domestic cat	*Felis catus*	FIV-fca	M25381
	Pallas’s cat	*Otocolobus manul*	FIV-oma	U56928
	Puma	*Puma concolor*	FIV-pco	EF455613
Simian immunodeficiency virus	Spot-nosed monkeys	*Cercopithecus nictitans*	SIV-gsn	AF468659
	Colobus monkey	*Colobus guereza*	SIV-col	AF301156
	Sykes' monkey	*Cercopithecus albogularis*	SIV-syk	L06042
	Dent's monkey	*Cercopithecus denti*	SIV-den	AJ580407
	Drill monkey	*Mandrillus leucophaeus*	SIV-drl	AY159321
	Green monkey	*Chlorocebus sabaeus*	SIV-agm-sab	U04005
	Mangabey	*Cercocebus torquatus*	SIV-rcm	HM803689
	Central chimpanzee	*Pan troglodytes*	SIV-cpz-ptt	AF103818
	Sooty mangabey	*Cercocebus atys atys*	SIV-smm	X14307
	Sun-tailed monkey	*Cercopithecus solatus*	SIV-sun	AF131870
	Mandrill	*Mandrillus sphinx*	SIV-mnd-1	M27470
Human immunodeficiency virus 1*	Human	*Homo sapiens*	HIV-1	AF033819
Human immunodeficiency virus 2	Human	*Homo sapiens*	HIV-2A	X05291
*Endogenous viruses*				
Rabbit endogenous lentivirus K	Leporids (rabbits & hares)		RELIK	[19]
Prosimian immunodeficiency virus 1	Mouse lemurs		PSIV1	[20]
Prosimian immunodeficiency virus 2	Dwarf lemurs		PSIV2	[21]
Mustelid endogenous lentivirus	Mustelids (subclade)		MELV	[22]
Dermopteran endogenous lentivirus	Colugos		DELV	[24]

^a^GenBank accession numbers are given for exogenous viruses. For endogenous lentivirus lineages consensus genome sequences were extracted from the publication shown

All retroviruses replicate via an obligate step in which a DNA copy of the viral genome is integrated into a host cell chromosome [5]. The integrated viral genome is flanked at either side by identical long terminal repeat (LTR) sequences (a form referred to as a ‘provirus’), each composed of functionally distinct U3, R and U5 regions. Occasionally, germline cells may be infected and subsequently go on to form viable progeny, so that integrated retroviral proviruses are vertically inherited as host alleles [6]. Such endogenous retroviruses (ERV) insertions are relatively common features in vertebrate genomes [7, 8]. Phylogenetic studies indicate that, following genome invasion, ERVs can increase their germline copy number through a variety of mechanisms, including active replication [9]. However, most ERV insertions are genetically fixed and highly degraded by germline mutation, reflecting their ancient origins. Frequently, deletion of the entire internal coding region occurs via homologous recombination between the provirus LTRs, so that only a ‘solo LTR’ sequence is left behind [10].

Even though their sequences are often extensively degraded, ERVs provide a valuable source of retrospective information about the long-term evolutionary interactions between retroviruses and their hosts [11]. For example, identification of orthologous ERV insertions in related species provides a robust means of deriving minimum age calibrations for retrovirus groups, based on host species divergence estimates (which are in part informed by the fossil record) [12]. More broadly, ERV sequences can be used to explore the long-term evolutionary history of ancient—presumably extinct—retrovirus groups [13, 14], and to inform our understanding of their interactions with host genes [15]. ERV sequences can even be used to guide the reconstitution of ancient retrovirus proteins so that their biological properties may be empirically investigated in vitro [16–18].

Lentiviruses have only rarely been incorporated into the germline of host species. However, a handful of Lentivirus-derived ERV lineages have now been identified (Table 1), and these sequences demonstrate that viruses clearly recognisable as lentiviruses circulated in mammals many millions of years ago. For example, rabbit endogenous lentivirus K (RELIK) insertions were found to occur at orthologous positions in the rabbit (*Oryctolagus cuniculus*) and hare (*Lepus europaeus*) genomes, demonstrating that genome invasion occurred prior to divergence of these species ~ 12 million years ago (Mya) [12, 19]. Endogenous lentiviruses have also been identified in lemurs (family Lemuridae) [20, 21]; mustelids (family Mustelidae) [22, 23]; and dermopterans (order Dermoptera—a group of arboreal gliding mammals native to Southeast Asia) [24–26]. Together, these sequences provide a range of minimum age calibrations in the Miocene epoch (23.5–5.3 Mya), based on host species divergence date estimates derived from the fossil record [11, 22, 25]. Widespread circulation among mammals is further supported by molecular clock-based age estimates that extend into the Eocene epoch (56–33.9 Mya) [24, 26].

In this study we perform comprehensive screening of published mammalian genomes and identify a previously unreported endogenous lentivirus lineage in the genome of the South African springhare (*Pedetes capensis*), demonstrating that lentivirus host range extends to rodents. Furthermore, through comparative and phylogenetic analysis, incorporating all available data, we provide broader insight into the origins and long-term evolutionary history of lentiviruses.

## Materials and methods

### Genome screening in silico

We used database-integrated genome screening (DIGS) [27] to derive a non-redundant database of lentivirus-derived ERV loci contained in published genome sequence assemblies. In DIGS, the output of systematic, sequence similarity search-based ‘screens’ is captured in a relational database. The DIGS tool [27] is a Perl-based framework in which the Basic Local Alignment Search Tool (BLAST) program suite (version 2.2.31+) [28] is used to perform systematic similarity searches of sequence databases (e.g., genome assemblies) and the MySQL relational database management system (MySQL Community Server version 8.0.30) is used to record and organise output data. WGS data of 431 mammalian species were obtained from the National Center for Biotechnology Information (NCBI) genome database [29] (Additional file 1: Table S1). Query polypeptide sequences were derived from representative lentivirus species (Table 1). DNA sequences in WGS assemblies that disclosed significant similarity to lentivirus queries (as determined by BLAST *e-*value) were classified via comparison to published retrovirus genome sequences (again using BLAST). Consensus genome sequences for endogenous lentivirus lineages were extracted from the supplementary material of associated publications, as follows: RELIK [19]; PSIV1 [20]; PSIV2 [21]; MELV [22]; DELV [24].

We compiled a set of endogenous lentivirus loci (Additional file 2: Table S2) by using structured query language) to filter screening the classified, non-redundant results of >130,000 searches, selecting matches based on their degree of similarity to lentivirus reference sequences, or the taxonomic characteristics of the species in which they occur. Using this approach we separated putatively novel lentivirus ERV loci from both (a) orthologs or paralogs of previously characterised lentivirus ERVs, and (b) non-lentiviral sequences that cross-matched to lentivirus probes due to shared ancestry (e.g., clade II ERVs) [30, 31]. We confirmed that putative novel lentivirus ERVs were indeed derived from lentiviruses (rather than other, related retroviruses) through phylogenetic and genomic analysis as described below.

### Phylogenetic and genomic analysis

Nucleotide and protein phylogenies were reconstructed using maximum likelihood (ML) as implemented in RAxML (version 8.2.12) [32]. Protein substitution models were selected via hierarchical maximum likelihood ratio test using the PROTAUTOGAMMA option in RAxML. To estimate the ages of solo LTRs we measured divergence from an LTR consensus sequence and applied a neutral rate calibration, as described by Subramanian et al*.* [33]. We used Se-Al (version 2.0) to visualise alignments and create consensus sequences [34].

## Results and discussion

We systematically screened WGS data representing 431 mammalian species (Additional file 1: Table S1) for endogenous lentivirus loci using similarity search-based approaches. As probes we used a comprehensive set of polypeptide products derived from the reference genomes shown in Table 1. We identified a total of 842 distinct lentivirus-derived ERV loci, most of which represented members of previously described lentivirus ERV lineages (Table 2, [35]). However, we also identified lentivirus-derived sequences in the genome of a species group in which they have not previously been described—rodents (order Rodentia).

**Table 2:** Endogenous lentivirus loci detected via screening

Organism	Common name	ERV lineage	Count
*Oryctolagus cuniculus*	European rabbit	RELIK	203
*Lepus americanus*	Snowshoe hare	RELIK	212
*Lepus timidus*	European hare	RELIK	121
*Sylvilagus bachmani*	Brush rabbit	RELIK	159
*Microcebus griseorufus*	Reddish-grey mouse lemur	PSIV1	20
*Microcebus mittermeieri*	Mittermeier's mouse lemur	PSIV1	20
*Microcebus murinus*	Grey mouse lemur	PSIV1	11
*Microcebus ravelobensis*	Golden-brown mouse lemur	PSIV1	21
*Microcebus tavaratra*	Northern rufous mouse lemur	PSIV1	20
*Cheirogaleus medius*	Fat-tailed dwarf lemur	PSIV2	1
*Mustela erminea*	Stoat	MELV	14
*Mustela putorius*	Ferret	MELV	18
*Neovison vison*	Mink	MELV	12
*Galeopterus variegatus*	Sunda colugo	DELV	6
*Pedetes capensis*	South African springhare	SpELV	17

RELIK, Rabbit endogenous lentivirus K; PSIV, prosimian immunodeficiency virus; MELV, Mustelid endogenous lentivirus; DELV, Dermopteran endogenous lentivirus; SpEL, springhare endogenous lentivirus

Matches to lentiviral Gag and Pol proteins were identified in WGS data of the South African springhare (*Pedetes capensis*), and the reverse transcriptase (RT) coding region encoded by one of these ERVs groups with previously described lentivirus species (Additional file 3: Fig. S1a). Initially, only four copies of Springhare endogenous lentivirus (SpELV) were identified in the *P. capensis* genome. However, we were able to identify the 5’ LTR of a partial provirus sequence by using sequences upstream from the *gag* ORF of the longest SpELV insertion (and spanning the region where a 5’LTR might be expected) as a query in BLASTn-based searches of the *P. capensis* genome assembly. This revealed the presence of a repetitive sequence showing the characteristic features of a retroviral LTR (i.e., ~ 500 nucleotides in length with terminal TG and CA dinucleotides) in the expected position upstream of the Gag ORF. Using this LTR sequence as input for screening enabled us to identify another 10 SpELV loci represented by solo LTR sequences (Table 3). We generated a consensus SpELV genome using all fourteen loci identified in our screen (Additional file 4: Fig. S2). We did not identify an envelope (*env*) gene associated with any SpELV insertions, nor did we identify any contigs containing complete proviruses with paired LTR sequences. Furthermore, because the longest provirus sequence we identified was truncated in *pol* we could not determine whether any accessory genes might have been encoded downstream of this gene. Nonetheless, the partial genome obtained in our analysis exhibits the characteristic features of lentivirus genomes, including (a) a primer-binding site specific for tRNA Lysine (Additional file 5: Fig. S3); (b) a Pro-Pol ORF expressed via -1 ribosomal frameshifting (Additional file 5: Fig. S3); (c) an adenine-rich (34%) genome (Additional file 6: Fig. S4) containing few CpG dinucleotides (0.29%); (d) a putative *trans*-activator response (TAR) element (Additional file 4: Fig. S2, Additional file 5: Fig. S3). We estimated the age of the SpELV lineage utilising a molecular clock-based approach in which divergence is calculated by comparing individual LTR sequences to an LTR consensus [33]. We obtained age estimates in the range of 8–18 Mya for SpELV loci (Table 3), consistent with an origin in the Middle Miocene.

**Table 3:** Springhare endogenous lentivirus loci

ERV locus^a^	Structure	Age (Mya) ^b^	Contig	Orientation	Start	End
SpELV.1-PedCap	LTR-Gag-Pol	8,750,000	VMDO01011022.1	+	63,971	68,252
SpELV.2-PedCap	LTR-Gag	12,500,000	VMDO01050306.1	+	2112	3978
SpELV.3-PedCap	Gag	*ND*	VMDO01082106.1	−	284	997
SpELV.4-PedCap	Gag	*ND*	VMDO01088624.1	−	445	1155
SpELV.6-PedCap	LTR	10,084,925	VMDO01001729.1	−	65,134	65,652
SpELV.14-PedCap	LTR	11,194,029	VMDO01006229.1	+	41,369	41,857
SpELV.15-PedCap	LTR	11,460,554	VMDO01001857.1	+	174,371	174,854
SpELV.16-PedCap	LTR	11,460,554	VMDO01001488.1	+	108,014	108,496
SpELV.17-PedCap	LTR	8,528,784	VMDO01000902.1	+	172,296	172,776
SpELV.18-PedCap	LTR	11,143,410	VMDO01003412.1	+	3534	4013
SpELV.21-PedCap	LTR	9,594,882	VMDO01035581.1	−	11,304	11,783
SpELV.24-PedCap	LTR	11,388,286	VMDO01046849.1	+	1434	1906
SpELV.25-PedCap	LTR	13,592,750	VMDO01015080.1	+	21,755	22,226
SpELV.28-PedCap	LTR	17,665,952	VMDO01001033.1	−	148,080	148,542
SpELV.32-PedCap	LTR	*ND*	VMDO01012895.1	+	13,691	14,124
SpELV.34-PedCap	LTR	*ND*	VMDO01003146.1	−	134,124	134,544
SpELV.35-PedCap	LTR	*ND*	VMDO01018267.1	+	34,458	34,865

^a^Loci were assigned unique identifiers (IDs) following a standard nomenclature system [42]. PedCap = *Pedetes capensis*. The ages of LTR-encoding elements was estimated by measuring divergence from an LTR consensus sequence and applying a neutral rate calibration, as described by Subramanian et al*.* [33]. *ND* = not done. SpELV = Springhare endogenous lentivirus

**Fig. 1:**
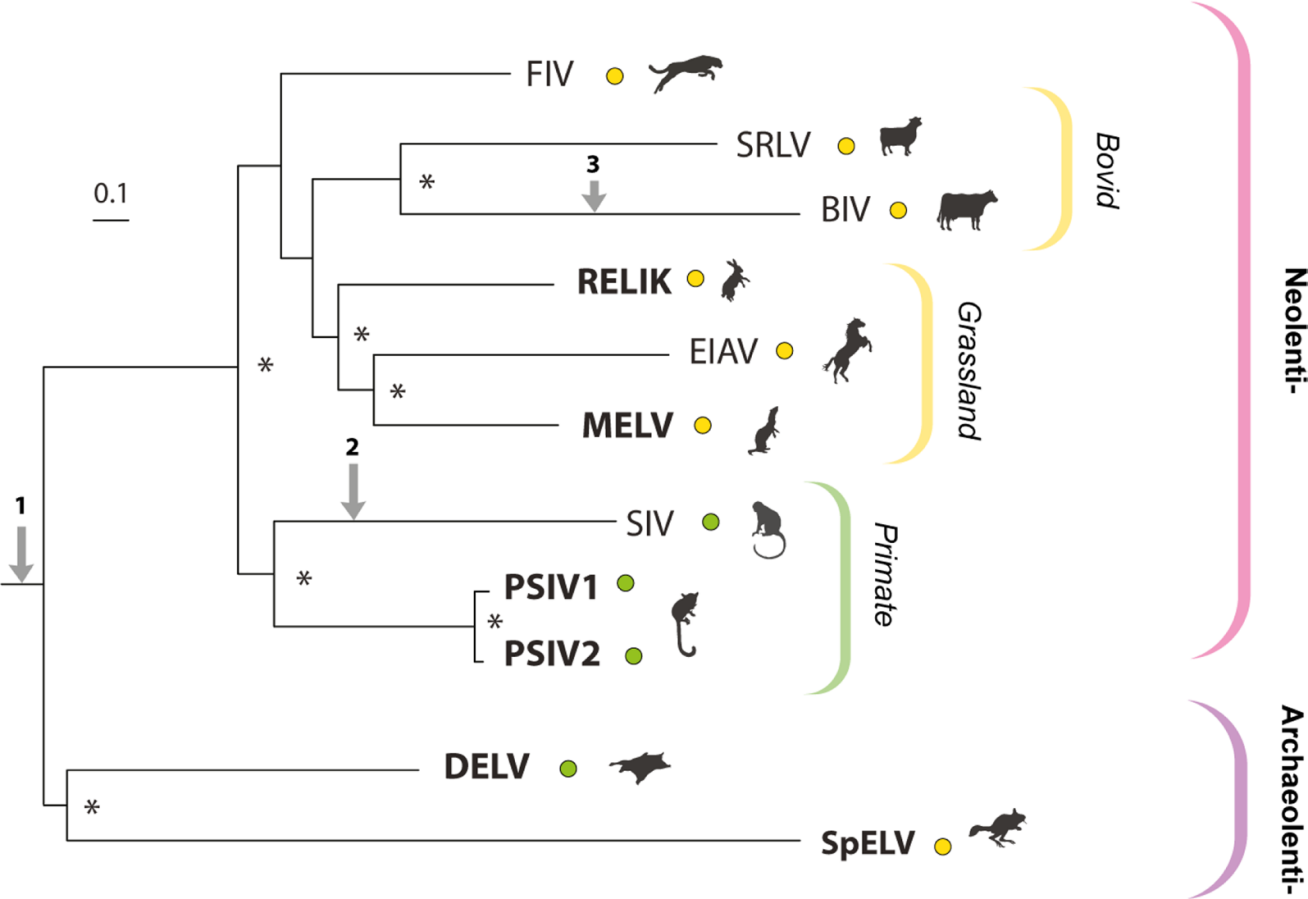
Phylogenetic relationships within the Lentivirus genus. Maximum likelihood phylogeny showing reconstructed evolutionary relationships between all known lentivirus species, including the extinct species represented by endogenous lentiviruses. Brackets to the left indicate proposed subgroupings within genus *Lentivirus*. Coloured circles adjacent virus taxa labels indicate the ecological characteristics of the associated host species (grassland-dwelling or arboreal) as shown in the key top right. The phylogeny is midpoint rooted for display purposes and was reconstructed using a multiple sequence alignment spanning 1405 amino acid residues of the Gag-Pol polyprotein and the RT-Rev substitution model [43]. The scale bar shows evolutionary distance in substitutions per site. Asterisks indicate nodes with bootstrap support > 70% (1000 replicates). Arrows indicate genome evolution events as follows (1) acquisition of *tat*, *rev* and *vif* genes (putatively); (2) loss of dUTPase; (3) partial loss of dUTPase. Abbreviations: FIV = Feline immunodeficiency virus; SRLV = small ruminant lentivirus; BIV = Bovine immunodeficiency virus; RELIK = Rabbit endogenous lentivirus type K; EIAV = equine infectious anaemia virus; MELV = Mustelidae endogenous lentivirus; SIV = Simian immunodeficiency virus; PSIV = Prosimian immunodeficiency virus; DELV = Dermopteran endogenous lentivirus; SpELV = springhare endogenous lentivirus

We used maximum likelihood-based phylogenetic approaches to reconstruct the evolutionary relationships between contemporary lentiviruses and the extinct lentiviruses represented by ERVs. Phylogenetic trees based on conserved regions of Gag-Pol clearly separate the Lentiviruses into two robustly supported subclades (Fig. 1). One (here labelled ‘Archaeolentivirus’) contains SpELV together with dermopteran endogenous lentivirus (DELV) which occurs in the germline of colugos (an unusual group of arboreal gliding mammals that are native to Southeast Asia) [24–26]. A second (here labelled ‘Neolentivirus’) contains all other endogenous lentivirus lineages and all known contemporary lentiviruses. We obtained relatively high support for internal branching relationships within the Neolentivirus clade–reconstructions support the existence of a distinct ‘primate’ group of neolentiviruses containing both simian and prosimian sub-lineages, and an ‘artiodactyl’ group incorporating both the bovine lentiviruses and the small ruminant lentiviruses. In addition, the primate lentiviruses group separately from all other neolentiviruses, which together constitute a ‘grasslands-associated’ clade comprised of lentiviruses that infect(ed) grassland-adapted host species (Fig. 1).

**Fig. 2:**
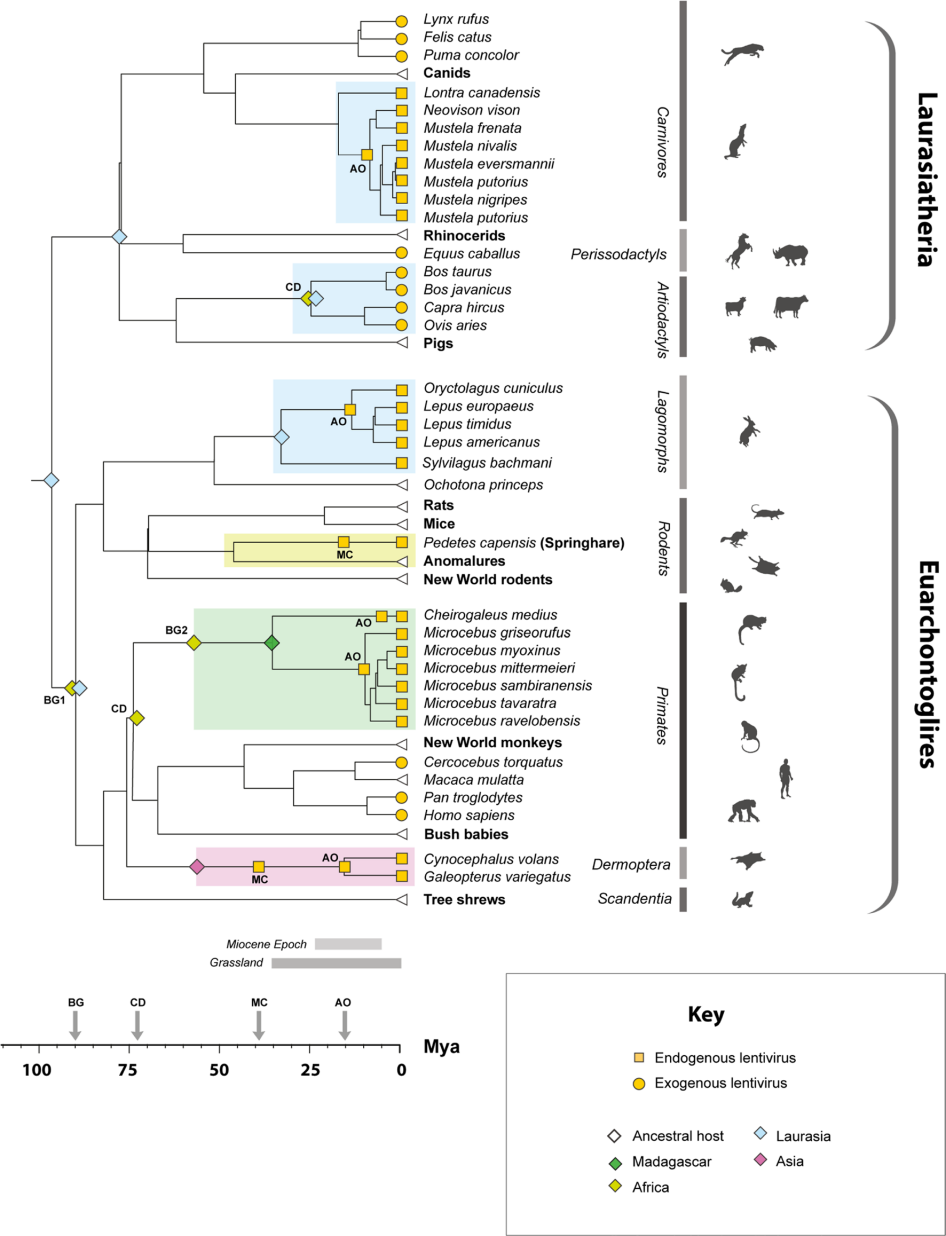
An updated timeline of lentivirus evolution. A time-calibrated phylogeny of mammalian species showing the known extent of association between lentiviruses and mammals, based on data obtained from TimeTree [44]. The scale bar shows time in millions of years before present. Brackets and bars to the right of taxa labels indicate host taxonomic groups. Yellow shapes on terminal nodes indicate that host species have known associations with endogenous lentiviruses (squares) or exogenous lentiviruses (circles). The timeframe of endogenous lentivirus presence in each mammalian lineage is indicated by shaded boxes behind clades, with colours indicating biogeographic associations of hosts within each clade following the key. White triangles at tree tips indicate host species or groups that have not yet been associated with any lentiviruses (endogenous or exogenous). Shapes on branches and internal nodes represent age calibrations for lentiviruses. Two-letter codes adjacent internal markers indicate the type of calibration being shown, as follows: AO = identification of an ancient ortholog; MC = application of a molecular clock to neutrally diverging sequences; CD = assumption of codivergence with hosts; BG = assumption of presence in biogeographic area inhabited by ancestor of species groups that are now biogeographically separated – note that this assumes no transfer between the respective regions identified by derived host species. Colours on diamond-shaped node markers indicate the known biogeographic range of ancestral hosts, following the key. The colonisation of Madagascar by lemurs (BG2) is thought to have occurred ~ 60 million years ago (Mya) [40, 41]

Plotting information about (a) known lentivirus distribution and (b) biogeographic range onto a time-calibrated phylogeny of boreoeutherian mammals provides some thought-provoking insights into lentivirus ecology and evolution (Fig. 2). Firstly, minimum age estimates established via orthology demonstrate that lentiviruses were widespread in the Miocene Epoch (i.e. ~ 20–5 Mya), both in terms of their host range and biogeographic distribution. It could potentially be significant that the diverse mammalian groups in which lentiviruses of the ‘grassland-associated’ clade are found (horses, bovids, mustelids and felids—see Fig. 1) all adapted to a grassland habitat during this period, in interconnected biogeographic areas (Laurasia and Africa) [36–38] (Fig. 2).

Regarding the ultimate origins of lentiviruses in mammals, molecular clock-based analyses of DELV insertions supports the presence of archaeolentiviruses in Asia (the only region where colugos occur) up to 60 Mya [26] – i.e., throughout most of the Cenozoic Era. The identification of SpELV shows that archeolentiviruses also circulated in springhare ancestors, which are found only in Africa. This raises the question of whether archeolentiviruses could have been present in the rodent-colugo ancestor that existed > 80 Mya [39] (Fig. 2). Such ancient origins would be consistent with the presence of primate lentivirus ancestors in the common ancestor of haplorrhine and strepsirrhine primates, and the arrival of lentiviruses in Madagascar ~ 60 Mya with founder populations of ancestral lemurs [40, 41] (Fig. 2). However, if extensive transmission between mammalian orders has occurred in the past, there would be other ways to account for observed lentivirus distributions without invoking such ancient origins.

## Conclusions

We describe a novel endogenous lineage in the genome of the South African springhare. The identification of SpELV demonstrates that lentivirus host range has historically extended to rodents.

## Supplementary Information

**Additional file 1.** **Table S1.** Whole genome sequence assemblies screened in this study.

**Additional file 2.** **Table S2.** Details of all endogenous lentivirus loci identified in this study.

**Additional file 3.** **Figure S1.** Phylogenetic and genomic characteristics of springhare endogenous lentivirus. (**a**) Maximum likelihood (ML) phylogeny based on an alignment of reverse transcriptase (RT) protein sequences and showing the reconstructed evolutionary relationships between lentiviruses and other retroviruses. Asterisks indicate nodes with bootstrap support > 70% (1000 replicates). The scale bar shows evolutionary distance in substitutions per site. (**b**) ML phylogeny showing reconstructed evolutionary relationships between SpELV long terminal repeat (LTR) sequences. Numbers next to nodes indicate bootstrap support values (1000 replicates). The scale bar shows evolutionary distance in substitutions per site. (**c**) Consensus genome structures of ancient lentiviral paleoviruses. DELV = Dermopteran endogenous lentivirus; RELIK = Rabbit endogenous lentivirus type K; Mustelidae endogenous lentivirus (MELV); BIV = Bovine immunodeficiency virus; SIV = Simian immunodeficiency virus; FIV = Feline immunodeficiency virus; Human immunodeficiency virus = HIV; Prosimian immunodeficiency virus = PSIV; RV = Retrovirus; LV = Leukemia virus.

**Additional file 4.** **Figure S2.** The SpELV consensus sequence. Inverted repeats present at the ends of the 5′ long terminal repeat (LTR) sequence are highlighted in light grey. Regions of nucleic acid secondary structure, the transactivation responsive (TAR) element and primer binding site (PBS) are highlighted in dark grey. The locations of the proteins encoded by the *gag* and *pol * genes were determined by homology to the DELV consensus sequence [24–26].

**Additional file 5.** **Figure S3.** The putative SpELV TAR (transactivation responsive region) element. Secondary structures were predicted using the MFOLD thermodynamic folding algorithm [45] and assessed by comparison to well-characterised examples in other lentiviruses.

**Additional file 6.** **Figure S4.** Nucleotide compositional bias in lentivirus genomes. Nucleotide composition of whole genomes of Lentiviruses were normalised to length and plotted as percentages using R in R Studio (version 4.2.1). Reference genome sequences for each virus correspond to those given in Table 1. Bovine immunodeficiency virus (BIV), Dermopteran endogenous lentivirus (DELV), Equine infectious anaemia virus American strain (EIAV_Am), Feline immunodeficiency virus (FIV), Human immunodeficiency virus 1 (HIV_1M), Mustelidae endogenous lentivirus (MELV), Prosimian immunodeficiency virus 2 (PSIV); Rabbit endogenous lentivirus type K (RELIK), Springhare endogenous lentivirus (SpELV), Small ruminant lentivirus A (SRLV_A); Adenine (A), Guanine (G), Cytosine (C), Thymine (T).

## Data Availability

All data are openly available in the Lentivirus-GLUE project hosted on GitHub: https://giffordlabcvr.github.io/Lentivirus-GLUE/.
