# Supplementary figures and images for: An endogenous lentivirus in the germline of a rodent
Source: Retrovirology. 2022 Dec 20;19:30. doi: 10.1186/s12977-022-00615-2 (PMC9768972; doi:10.1186/s12977-022-00615-2)

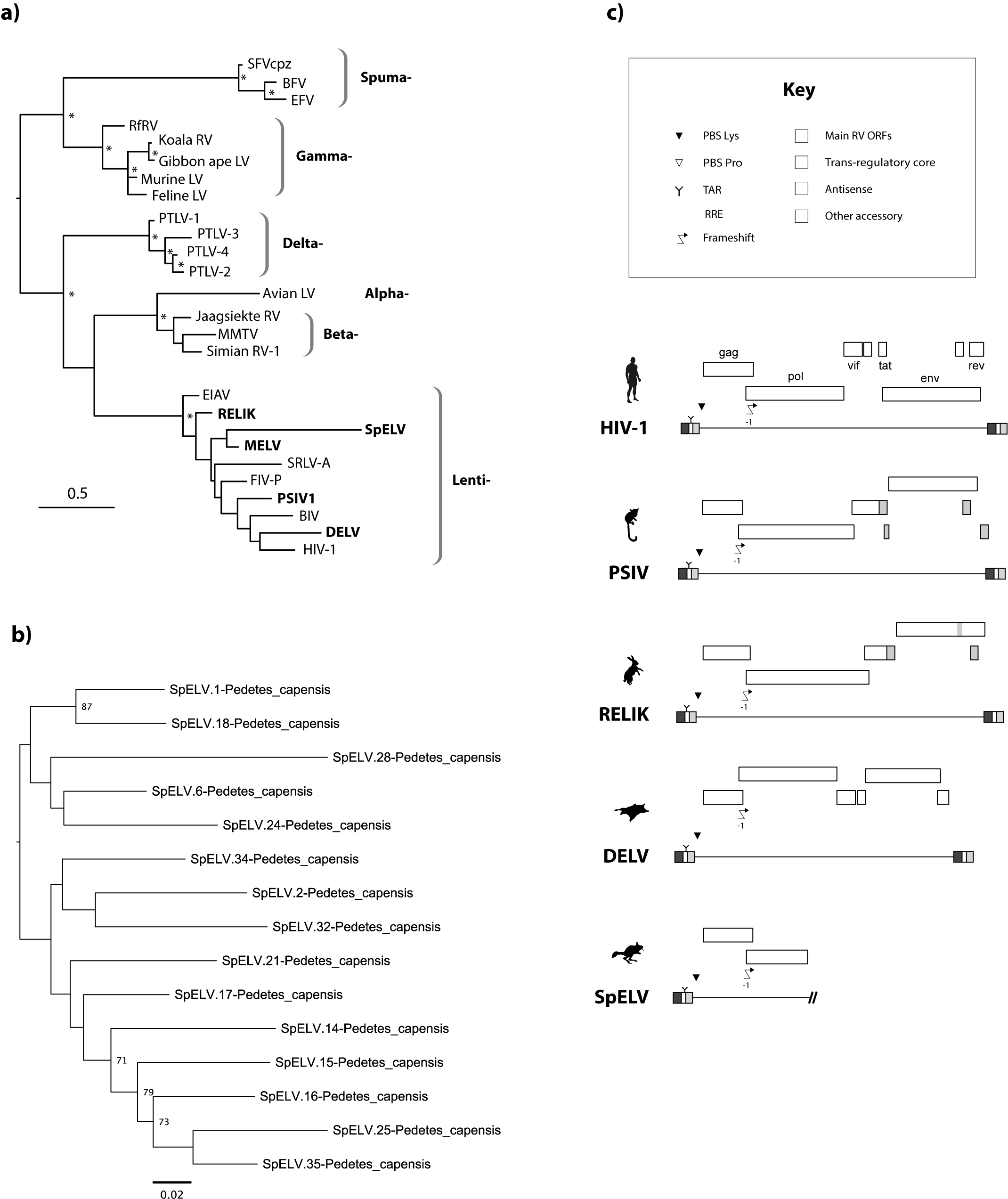

Supplement: Supplementary file 3 — Additional file 3. Figure S1. Phylogenetic and genomic characteristics of springhare endogenous lentivirus. (a) Maximum likelihood (ML) phylogeny based on an alignment of reverse transcriptase (RT) protein sequences and showing the reconstructed evolutionary relationships between lentiviruses and other retroviruses. Asterisks indicate nodes with bootstrap support > 70% (1000 replicates). The scale bar shows evolutionary distance in substitutions per site. (b) ML phylogeny showing reconstructed evolutionary relationships between SpELV long terminal repeat (LTR) sequences. Numbers next to nodes indicate bootstrap support values (1000 replicates). The scale bar shows evolutionary distance in substitutions per site. (c) Consensus genome structures of ancient lentiviral paleoviruses. DELV = Dermopteran endogenous lentivirus; RELIK = Rabbit endogenous lentivirus type K; Mustelidae endogenous lentivirus (MELV); BIV = Bovine immunodeficiency virus; SIV = Simian immunodeficiency virus; FIV = Feline immunodeficiency virus; Human immunodeficiency virus = HIV; Prosimian immunodeficiency virus = PSIV; RV = Retrovirus; LV = Leukemia virus. [file 12977_2022_615_MOESM3_ESM.tif]

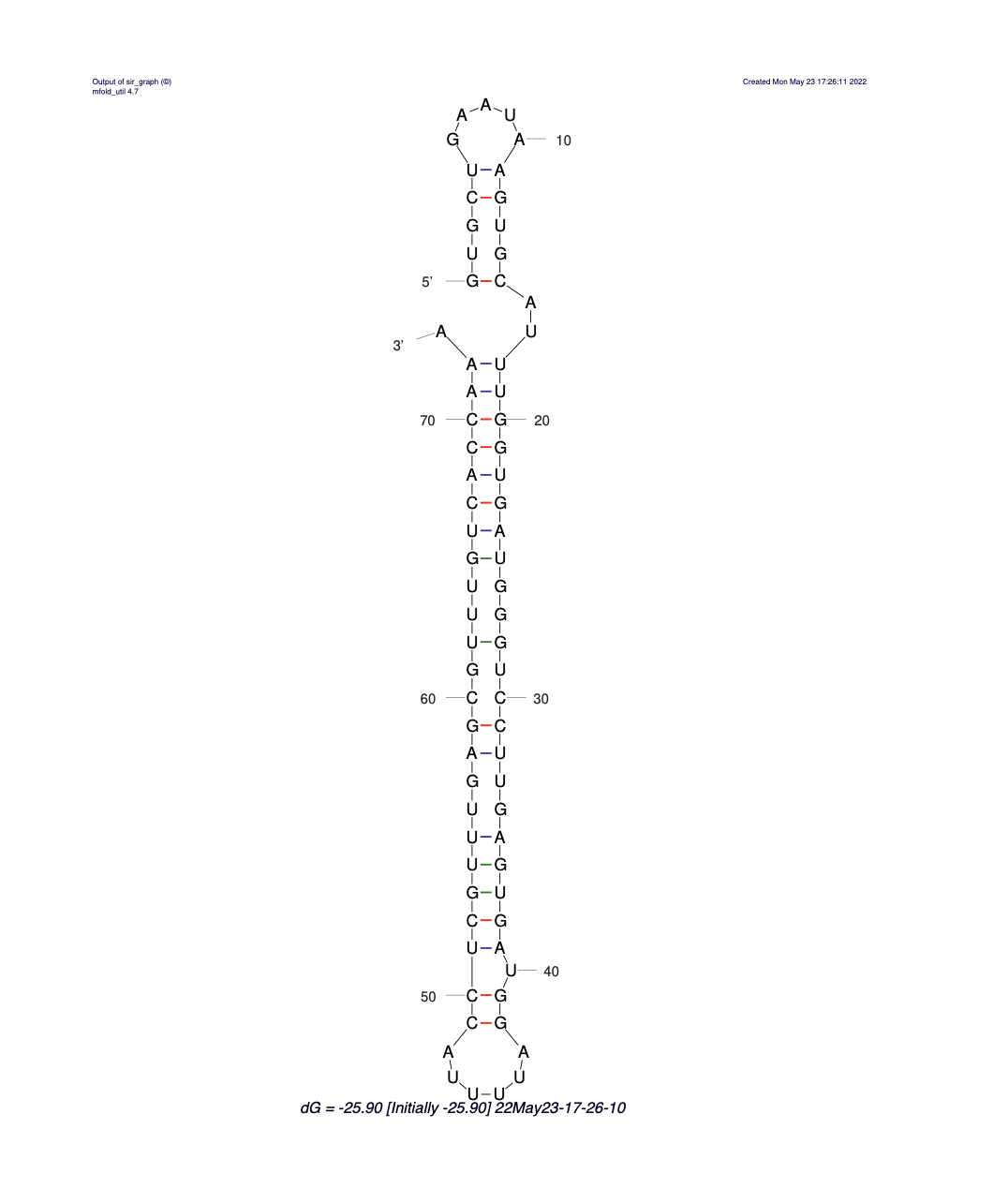

Supplement: Supplementary file 5 — Additional file 5. Figure S3. The putative SpELV TAR (transactivation responsive region) element. Secondary structures were predicted using the MFOLD thermodynamic folding algorithm [45] and assessed by comparison to well-characterised examples in other lentiviruses. [file 12977_2022_615_MOESM5_ESM.png]

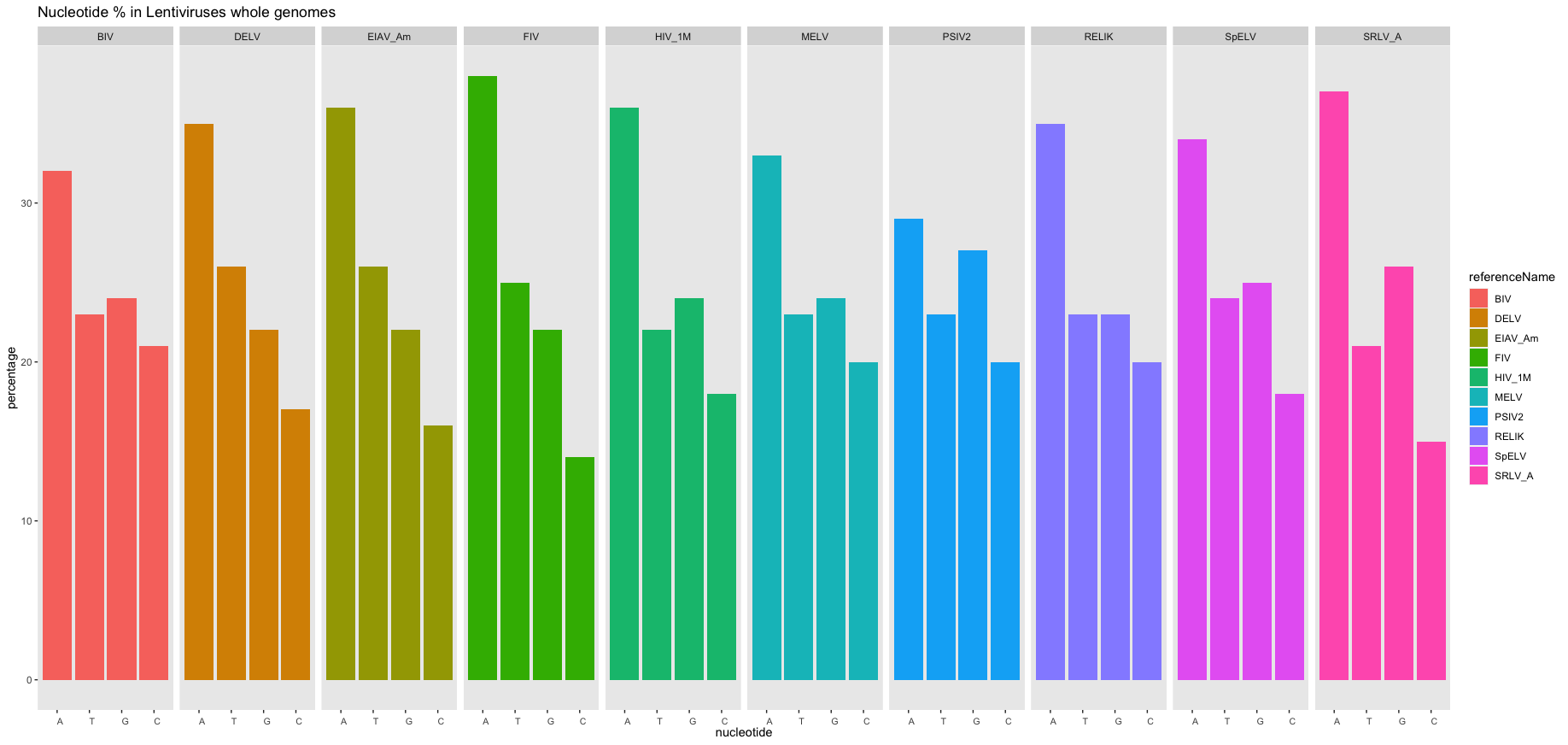

Supplement: Supplementary file 6 — Additional file 6. Figure S4. Nucleotide compositional bias in lentivirus genomes. Nucleotide composition of whole genomes of Lentiviruses were normalised to length and plotted as percentages using R in R Studio (version 4.2.1). Reference genome sequences for each virus correspond to those given in Table 1. Bovine immunodeficiency virus (BIV), Dermopteran endogenous lentivirus (DELV), Equine infectious anaemia virus American strain (EIAV_Am), Feline immunodeficiency virus (FIV), Human immunodeficiency virus 1 (HIV_1M), Mustelidae endogenous lentivirus (MELV), Prosimian immunodeficiency virus 2 (PSIV); Rabbit endogenous lentivirus type K (RELIK), Springhare endogenous lentivirus (SpELV), Small ruminant lentivirus A (SRLV_A); Adenine (A), Guanine (G), Cytosine (C), Thymine (T). [file 12977_2022_615_MOESM6_ESM.tiff]
