# Supplementary material for: An endogenous lentivirus in the germline of a rodent
Source: Retrovirology. 2022 Dec 20;19:30. doi: 10.1186/s12977-022-00615-2 (PMC9768972; doi:10.1186/s12977-022-00615-2)
Supplement: Supplementary file 4 — Additional file 4. Figure S2. The SpELV consensus sequence. Inverted repeats present at the ends of the 5′ long terminal repeat (LTR) sequence are highlighted in light grey. Regions of nucleic acid secondary structure, the transactivation responsive (TAR) element and primer binding site (PBS) are highlighted in dark grey. The locations of the proteins encoded by the gag and pol genes were determined by homology to the DELV consensus sequence [24–26]. [file 12977_2022_615_MOESM4_ESM.pdf]

```
       I.R.
1      TGTTAGCCCTTGGGGAACTTTTGTCACCAGGACCTGCAACATCTGAAGAATTGAGAAGATTGCTAAATCAATCTGATGGA  80

81     CTTGCGGACAGCCGGGGGTGGAGTCACTTGGGGAGAAATGGGTCATATCCTGCAGAAGAAGTAAGCTAAACTGGAGATTG  160
                                                            Transactivation response (TAR)element
161    AGATTTGCAGAAGTGGCAAGCCAGCCTGATAGATAGTGCTGAATAAGTGCATTTGGTGATGGGTCCTTGAGTGATGGATT  240

241    TTACCTCGTTTGAGCGTTTGTCACCAAATACTCTGTCAGCAAATTGCCATGCTTGATTGACATATCCGTTTGTTTTTTTT  320

321    GTCTATATATACTTTGAGGACTGCTTCATGGGGAGAGACCTTAGGTCTATCCTCGTGACCCAATAAAGCATTGCAGAACT  400
                                                                                  I.R.
401    AGACACTGGTCTCCCGAGAATTTTGTCTAGGTTCTGGGCAGTAAGCTTCCTCCTCGGCGGGTCTCTTCACCCTTTGGGTG  480
                                   Primer binding site
481    CACTGGGGGCACGGGAGTTTACAGGTGGCGCCCAGTGTGGGGCTCCATAGGGCTTGAGAAGGGGCTCAGGAAGGGGAGTC  560

561    CACACTCTTGCTAGATGTCCTCAGAAGGCTGTCCTGAGAGGCCAATAGACAGCTCCCCAAAGCTGAGAGAAACTAAAAGC  640

                                                                                M  S  N
641    GAAACTAACAGGTGCAGCCCAGCACAGGCAGCCAATGGACGCATCACTGGAAAGAGGAAGCAGTGAGTGAAAATGAGCAA  720

        G  S  S  L  G  K  D  L  R  E  L  E  E  K  F  S  K  E  L  T  P  K  V  K  G  N  L
721    TGGATCCAGCTTGGGGAAAGATCTGAGAGAGCTTGAGGAAAAATTTAGTAAGGAGCTCACACCTAAAGTGAAAGGGAATT  800

        K  I  L  T  K  V  A  Q  V  E  G  G  I  Y  D  P  G  Y  L  G  Y  V  F  T  A  I
801    TAAAGATCTTAACTAAGGTAGCTCAAGTGGAAGGTGGCATCTATGACCCAGGGTATTTAGGATATGTCTTCACAGCCATT  880

       E  D  F  L  L  Q  T  E  A  A  C  Q  G  I  L  L  S  G  H  L  L  E  K  G  M  I  I
881    GAGGATTTTTTGTTGCAGACTGAAGCCGCATGCCAGGGCATACTTTTGTCTGGACATTTGCTGGAAAAAGGGATGATTAT  960

        K  L  V  T  F  L  L  E  Q  E  K  E  K  L  A  R  A  W  M  V  F  Y  A  V  V  I  Q
961    TAAACTAGTAACTTTCTTGCTAGAACAGGAGAAGGAAAAGCTAGCAAGAGCATGGATGGTGTTTTATGCAGTAGTGATTC  1040

        G  I  P  L  R  Q  R  G  L  L  V  K  H  G  M  M  W  R  R  P  R  A  R  S  V  R
1041   AAGGAATTCCGTTAAGACAGAGAGGGCTGCTTGTCAAGCATGGAATGATGTGGCGGAGGCCAAGGGCCCGGTCTGTCAGG  1120

       S  E  V  Q  G  Q  E  E  A  S  V  N  P  V  T  R  V  P  Q  G  G  P  V  P  I  K  F
1121   TCTGAAGTACAAGGACAAGAGGAGGCATCAGTAAACCCTGTAACTAGAGTACCACAGGGAGGTCCAGTGCCTATAAAATT  1200

        P  L  K  E  L  T  R  I  A  S  V  T  V  E  H  G  S  L  S  D  P  V  Q  H  H  L  L
1201   TCCATTGAAGGAGTTGAC|CAGAATAGCTTCTGTAACAGTTGAACATGGTTCCCTCTCAGATCCAGTTCAACACCATTTAT  1280
              Matrix <----|----> Capsid
        M  L  S  T  A  D  L  T  P  G  D  W  M  T  V  F  S  A  M  Q  G  N  G  A  I  K
1281   TAATGCTGTCAACTGCTGATCTGACTCCAGGAGACTGGATGACTGTGTTTTCAGCAATGCAAGGAAATGGGGCAATAAAG  1360

       T  G  I  Q  G  L  I  A  Q  K  M  E  E  D  E  E  A  N  G  P  G  S  S  Q  P  I  I
1361   ACAGGAATACAAGGGTTAATAGCTCAAAAAATGGAAGAGGATGAGGAAGCAAATGGACCAGGCTCATCACAGCCTATTAT  1440

       G  T  N  M  T  A  A  Q  Q  A  S  D  Q  Q  A  P  H  Y  K  L  F  M  Q  W  I  L  D
1441   AGGGACAAATATGACAGCTGCCCAGCAGGCTAGTGATCAGCAGGCACCTCACTACAAACTATTTATGCAGTGGATCTTAG  1520

        T  C  Q  Q  L  R  E  K  V  G  G  A  L  I  P  P  T  R  I  L  Q  E  P  K  E  P
1521   ATACATGTCAGCAACTGAGAGAAAAAGTGGGAGGTGCTCTGATACCCCCTACCAGGATTTTGCAAGAGCCAAAGGAGCCC  1600

        Y  G  D  F  T  D  Q  L  H  V  A  I  E  K  L  T  M  S  Q  E  L  K  E  E  L  K  D
1601   TATGGCGACTTCACAGATCAACTCCATGTGGCCATAGAAAAGTTAACAATGAGTCAAGAGTTGAAGGAAGAATTAAAGGA  1680

        R  L  S  V  D  N  T  N  G  D  C  K  R  A  L  G  K  I  E  Y  G  D  P  L  V  D
1681   CAGGTTATCAGTAGATAACACCAATGGTGATTGCAAGAGAGCATTAGGAAAAATTGAGTATGGAGATCCACTAGTGGACA  1760

       K  L  K  S  C  Q  N  V  G  T  L  T  W  K  K  A  L  M  A  E  T  E  A  P  K  N  N
1761   AACTAAAATCATGTCAGAATGTAGG|AACACTGACATGGAAAAAGGCACTTATGGCGGAAACCGAAGCACCTAAGAACAAT  1840
                      Capsid <----|----> Nucleocapsid
        Q  R  V  I  Q  P  T  S  R  K  I  I  C  F  K  C  G  T  A  G  H  I  K  R  N  C  R
1841   CAGAGGGTAATACAACCTACCAGCAGGAAGATTATTTGTTTTAAATGTGGTACAGCTGGACACATAAAAAGGAACTGTAG  1920

        K  G  S  Q  D  R  R  E  P  N  L  C  L  I  C  K  K  E  K  R  W  T  S  Q  C  P
1921   GAAAGGATCACAGGACAGGAGAGAGCCAAATCTCTGTCTGATTTGCAAGAAAGAGAAGCGCTGGACATCTCAATGTCCAC  2000

       Q  E  K  N  *  H  G  G  T  Q  K  G  T  Q  F  P  S  M  D  S  K  I  V  P  *  P  T
2001   AGGAAAAAAACTAGCATGGGGGGACTCAGAAGGGCACTCAGTTCCCTAGCATGGACAGTAAAATTGTGCCCTGACCAACC  2080
```

E I E K I R T L K Y R P C L L I Q T P L E E

* V F I G N R K D K N S Q V

2081 TAAGTCTTTATAGGAAATAGAAAAGATAAGAACTCTCAAGTATAGACCATGTTTATTAATACAAACTCCTTTGGAAGAAA 2160

Protease active site

I N S L M D T G A D L R I L G E Q I K V D H Y P M G A

2161 TTAATTCACTGATGGACACAGGAGCAGACCTAAGAATCTTAGGAGAACAGATAAAAGTAGATCATTACCCAATGGGAGCT 2240

S I K V T G I G D S Q K F Q F Y L Y G V D I R G R F G

2241 TCCATAAAAGTAACTGGAATAGGAGACTCCCAGAAATTCCAATTCTATCTATATGGTGTAGATATTAGAGGAAGGTTTGG 2320

N R M A H M P G T M D L L G * D A L E I L G I R L V

2321 GAATAGGATGGCACATATGCCAGGAACTATGGATTTATTAGGGTGAGATGCTCTAGAAATACTAGGCATAAGGTTAGTAG 2400

G A V L S T K L Q P V M P A F K P N A K F P K L K Q W

2401 GAGCTGTACTGTCTACAAAATTACAGCCTGTCATGCCAGCTTTTAAGCCAAATGCCAAGTTTCCAAAACTCAAACAGTGG 2480

Pro ← → RT

P I S A E K L K D I K S I T D S L L S E N K I R K A A

2481 CCGATTTCAGCCGAGAAGCTAAAAGACATAAAGTCAATAACTGACTCCTTGCTTTCTGAAAATAAGATTAGAAAAGCGGC 2560

P G N P W N T P C F V I K K R D G K T F R L S M D F

2561 CCCAGGAAATCCATGGAACACTCCATGTTTTGTTATTAAGAAAAGGGATGGAAAAACTTTTAGATTATCAATGGACTTTA 2640

K Q L N E C T E Y V V A T N P G L P H P S G I L R M H

2641 AACAACTAAATGAATGTACTGAATATGTGGTGGCAACCAACCCAGGCTTACCTCATCCATCAGGCATCCTTAGAATGCAC 2720

K F H V L L D M A N A Y F T V P I A E E F R P Y T A F

2721 AAATTTCATGTCTTATTAGATATGGCCAATGCCTACTTTACTGTACCCATTGCTGAGGAGTTCAGGCCCTACACTGCATT 2800

T V P Q I N M V G L G D R Y E W C C L P K G W N G S P

2801 TACAGTACCTCAGATCAACATGGTAGGACTGGGAGATAGATATGAATGGTGTTGTTTACCAAAAGGCTGGAATGGGAGCC 2880

E T F Q S T L R P I I A V I E R R K S K A V S I I T

2881 CAGAAACTTTTCAATCTACTCTAAGACCTATTATAGCAGTTATAGAAAGAAGGAAGTCTAAAGCAGTCTCCATAATTACT 2960

RT active site

Y M D D I L I S G E T E A Q V E R I K L L T E E F Q K

2961 TATATGGATGACATCTTAATCTCAGGAGAAACAGAGGCCCAAGTGGAGAGAATAAAACTACTCACAGAAGAATTTCAGAA 3040

W G F E L P P D K Q Q R G K N I E R V L G Y C L T D

3041 GTGGGGTTTTGAGTTGCCTCCAGATAAGCAACAGAGAGGAAAGAATATAGAGAGAGTCTTAGGTTATTGCCTTACTGATG 3120

E G W K P T N M E L R K E E I Q T L H D V Q V V R E T

3121 AGGGATGGAAACCCACAAACATGGAACTAAGGAAGGAGGAAATACAGACTTTACATGATGTACAAGTTGTTAGGGAAACT 3200

T M V R D W V P I D L T P I H Y L L R G D Q D L L S P

3201 ACAATGGTTAGGGACTGGGTACCTATAGATCTTACTCCCATACATTACTTGCTGCGAGGAGATCAAGACCTTCTCAGTCC 3280

Q K A T P E V N R L L Q E V D Q K I K S E L E R G R

3281 ACAAAAAGCCACCCCTGAAGTGAATAGGCTATTACAGGAAGTAGATCAAAAAATAAAGTCAGAATTGGAGAGAGGAAGGA 3360

D P Q K D L E G S W D T L G V T I H Q G K V I L S W

3361 TAGATCCACAAAAGGACTTAGAGGGATCTTGGGATACTCTAGGAGTTACCATCCATCAGGGGAAGGTGATTCTTAGTTGG 3440

A P F T F P N G T V D L L S L L D S S V D K V Q M F E

3441 GCACCATTCACGTTCCCAAATGGAACAGTGGATTTGCTCTCATTACTAGACAGTTCAGTGGACAAGGTTCAGATGTTTGA 3520

```
      L  L  R  Y  G  Y  E  S  K  I  I  N  Q  S  G  S  *  K  E  L  K  S  L  Q  M  Q
3521  ATTATTAAGATATGGATATGAGTCCAAGATAATAAACCAGTCAGGGTCCTAGAAAGAATTAAAAAGTTTACAAATGCAGG  3600
      D  V  W  P  T  R  W  T  Y  K  K  F  I  C  K  N  G  Q  G  W  T  I  G  L  S  N  L
3601  ATGTGTGGCCTACAAGATGGACATATAAGAAGTTTATTTGTAAAAATGGTCAGGGTTGGACTATAGGATTATCCAATCTA  3680
      L  Q  I  R  R  I  E  K  T  P  I  V  G  G  E  T  V  Y  T  D  A  S  R  L  R  K  T
3681  CTTCAGATTAGGAGGATTGAAAAAACACCTATTGTAGGAGGAGAGACTGTCTATACAGATGCATCGAGGTTGCGGAAAAC  3760
      N  H  K  R  I  A  W  Y  N  T  T  T  G  D  T  H  S  M  E  V  S  T  E  T  G  H
3761  TAACCACAAGAGAATAGCTTGGTACAACACAACCACGGGAGACACACATAGTATGGAAGTAAGCACAGAAACAGGACATG  3840
      A  *  Q  A  E  L  L  A  I  I  G  V  L  I  N  H  P  R  S  L  N  I  V  T  H  S  K
3841  CATGACAAGCAGAGCTCTTAGCAATTATAGGAGTACTAATTAATCATCCTAGGTCACTAAATATAGTAACACATAGCAAA  3920
      Y  I  A  A  F  L  P  K  I  G  G  H  E  R  N  N  L  W  Q  E  V  I  A  M  L  A  E
3921  TATATTGCAGCTTTTCTACCAAAAATAGGAGGACATGAGAGAAATAACCTATGGCAAGAAGTAATAGCCATGCTGGCAGA  4000
      R  V  K  Q  R  Y  R  T  F  V  S  W  V  P  G  H  S  E  V  Q  E  M
4001  AAGGGTAAAGCAAAGATATAGAACATTTGTTTCTTGGGTTCCTGGACACAGTGAGGTCCAGGAAATGA  4068
```
